# Targeting nucleotide metabolism: a promising approach to enhance cancer immunotherapy

**DOI:** 10.1186/s13045-022-01263-x

**Published:** 2022-04-27

**Authors:** Huai-liang Wu, Yue Gong, Peng Ji, Yi-fan Xie, Yi-Zhou Jiang, Guang-yu Liu

**Affiliations:** Key Laboratory of Breast Cancer in Shanghai, Department of Breast Surgery, Department of Oncology, Fudan University Shanghai Cancer Center, 270 Dongan Road, Xuhui District, Shanghai, 200032 People’s Republic of China

## Abstract

Targeting nucleotide metabolism can not only inhibit tumor initiation and progression but also exert serious side effects. With in-depth studies of nucleotide metabolism, our understanding of nucleotide metabolism in tumors has revealed their non-proliferative effects on immune escape, indicating the potential effectiveness of nucleotide antimetabolites for enhancing immunotherapy. A growing body of evidence now supports the concept that targeting nucleotide metabolism can increase the antitumor immune response by (1) activating host immune systems via maintaining the concentrations of several important metabolites, such as adenosine and ATP, (2) promoting immunogenicity caused by increased mutability and genomic instability by disrupting the purine and pyrimidine pool, and (3) releasing nucleoside analogs via microbes to regulate immunity. Therapeutic approaches targeting nucleotide metabolism combined with immunotherapy have achieved exciting success in preclinical animal models. Here, we review how dysregulated nucleotide metabolism can promote tumor growth and interact with the host immune system, and we provide future insights into targeting nucleotide metabolism for immunotherapeutic treatment of various malignancies.

## Introduction

Nucleotides are the main building blocks of genetic materials and are composed of purines (adenine and guanine) and pyrimidines (thymine, uracil, and cytosine). They are essential substances for the biosynthesis of DNA and RNA, cell signaling, enzyme regulation, and metabolism. Cancer cells must synthesize and utilize large amounts of energy and nucleotides for DNA and RNA, and upregulated de novo nucleotide metabolism enables cells to proliferate rapidly; therefore, nucleotide metabolism is a potential target for cancer treatment. Although numerous efforts to target this attractive metabolic pathway have been reported, the key enzymes and regulatory mechanisms involved in nucleotide metabolism remain unclear. All classical antitumor drugs inhibiting nucleotide synthesis are based on analogs of tumor nucleotide metabolites and have previously served as chemotherapies in cancer treatment [1, 2]. However, due to their lack of specificity for tumor cell nucleotide metabolism, these drugs also inhibit the metabolic processes of normal cells, causing serious side effects [3, 4]. Therefore, more in-depth study of the regulatory processes of nucleotide metabolism has important theoretical and clinical significance. Moreover, recent studies have shown that abnormal nucleotide metabolism not only accelerates the development of tumors but also alters the normal immune response in the tumor microenvironment (TME), indicating the potential effectiveness of targeting nucleotide metabolism to enhance immunotherapy [5–7]. This review provides an overview of nucleotide metabolism and its role in cancer and emphasizes that nucleotide metabolism is a therapeutic target not only for chemotherapy but also for enhancing the efficacy of cancer immunotherapy.

### Nucleotide biosynthesis and degradation

Nucleotide metabolism includes nucleotide biosynthesis and degradation to maintain nucleotide homeostasis [8]. Proliferating cells acquire nutrients (mainly glucose, glutamine, and CO_2_) to generate energy to drive anabolism of nucleic acids, while nucleotides also need to be replenished at certain rates consistent with nucleotide biosynthesis [9]. Although nucleotides can be taken up via salvage pathways, the de novo biosynthesis pathway remains the main pathway through which most dividing cells synthesize nucleotides and their related metabolites [10, 11].

De novo nucleotide metabolism is regulated by several critical metabolic genes and encoded enzymes (Table 1). These essential metabolic enzymes play important roles in maintaining nucleotide biosynthesis [12]. Purine biosynthesis, in which purine nucleotides are synthesized directly by the addition of a pyrophosphate at C-1 of the ribose sugar, differs from pyrimidine biosynthesis in many ways [13]. Purine biosynthesis begins with ribose-5-phosphate converted to phosphoribosyl pyrophosphate (PRPP), and several ATP equivalents are required to activate PRPP [9]. The enzyme involved in this step is PRPP synthetase, encoded by the gene *PRPS* (Table 1). The rate-limiting step in this pathway is the second step, wherein PRPP is catalyzed by PPAT to bind with glutamine, causing the formation of 5-phosphoribosylamine along with the release of pyrophosphate [14–16]. The next steps characterized as ATP-dependent include several reactions in which inosine monophosphate (IMP) is converted and biosynthesized from 5-phosphoribosylamine, and glycinamide ribonucleotide transformylase (GART) plays a key role in maintaining its biosynthesis [17]. IMP serves as a precursor to adenosine monophosphate (AMP) and guanosine monophosphate (GMP) synthesis [17]. During the synthesis of AMP and GMP from IMP, adenylosuccinate synthetase (ADSS) and Inosine monophosphate dehydrogenase (IMPDH) are essential catalysts of the conversion of IMP into succinyl adenosine 5'-monophosphate (sAMP) and xanthosine monophosphate (XMP) through several kinetic intermediates [18–20].

**Table 1:** Key metabolic genes and related metabolites from the nucleotide metabolism in cancer immunity

Key metabolic genes	Involved metabolic module	Metabolic substrate	Metabolic product	Therapeutic agents	References
*Nucleotide metabolism*					
NT5E (CD73)	Purine nucleobase metabolism, pyrimidine nucleobase metabolism, adenosine biosynthesis, AMP catabolic process, DNA metabolic process, purine nucleotide biosynthesis	Phosphated ribonucleoside	Ribonucleoside	Oleclumab, AB680, APCP	[21, 22]
ENTPD1(CD39)	Purine metabolism, Pyrimidine metabolism	Phosphated ribonucleoside	Phosphated ribonucleoside	TTX-030, IPH5201	[21, 23, 24]
PNP	Purine-nucleoside phosphorylase activity	Ribonucleoside	Phosphated ribonucleoside	Forodesine	[25–27]
*Purine metabolism*					
ADSS	Adenine ribonucleotide biosynthesis	IMP	ADP, ATP		[28]
ADA	Purine metabolism	Adenosine	Inosine	Elapegademase, pentostatin	[29]
XDH	Purine metabolism	Hypoxanthine, Xanthine	Xanthine, uric acid	Allopurinol, amflutizole	[30]
PPAT	5-Phosphoribosylamine biosynthesis	PRPP	5-Phosphoribosylamine and pyrophosphate	D-pantetheine 4'-phosphate	[31]
PRPS	PRPP biosynthesis	Ribose 5P	PRPP		[32, 33]
GART	Inosine monophosphate biosynthesis	PRPP	IMP	Lometrexol sodium, Pelitrexol, Pemetrexed	[34, 35]
IMPDH	Guanine ribonucleotide biosynthesis	IMP	GDP, GTP	Merimepodib, mizoribine, mycophenolic acid	[36–40]
*Pyrimidine metabolism*					
CAD	Uridine monophosphate biosynthesis	Glutamine	UMP		[41, 42]
DHODH	Uridine monophosphate biosynthesis	Dihydroorotate	Orotate	Brequinar sodium, leflunomide, and teriflunomide	[43–46]
UMPS	Uridine monophosphate biosynthesis	Glutamine	UMP		[8, 42, 47]
DPYD	Pyrimidine degradation	Uracil, thymine	Beta-alanine, 3-aminoisobutanoate	Eniluracil, Gimeracil	[48–50]
CDD	Uridine monophosphate synthesis	Cytidine, Deoxycytidine	Uridine, Deoxyuridine		[51, 52]

*APCP* adenosine 5'-(alpha, beta-methylene) diphosphate

In the de novo pyrimidine biosynthesis pathway, the pyrimidine ring structure is assembled through a 6-step process with L-glutamine and L-aspartate as precursors, which are transformed into dihydroorotate in the initial steps [9]. The trifunctional proteins carbamoyl phosphate synthetase, aspartyl transcarbamoylase, and dihydroorotase (CAD) are associated with the enzymatic activities of the first three reactions [41, 42]. As another well-known rate-limiting enzyme of pyrimidine biosynthesis, dihydroorotate dehydrogenase (DHODH) catalyzes dihydroorotate into orotate and derives mitochondrial electron transport and oxygen consumption [43–45, 53]. The final two steps of the de novo pyrimidine biosynthetic pathway are catalyzed by uridine monophosphate synthetase (UMPS), a bifunctional enzyme that includes orotate phosphoribosyltransferase and orotidine monophosphate (OMP) decarboxylase [8, 42, 47]. The first reaction is initiated from orotate to form orotidine-5P, while orotidine-5P is converted into uridine-5-phosphate in the second step [54, 55]. Uridine-5-phosphate constitutes the building block of the subsequent reactions of pyrimidine biosynthesis [9].

The common pathways of both purine and pyrimidine nucleotide biosynthesis include several reactions. Generally, the transformation and homeostasis between nucleoside triphosphate and nucleoside monophosphate are controlled by ecto-nucleoside triphosphate diphosphohydrolase-1 (ENTPD1) (Table 1). ENTPD1 (also known as CD39) and ecto-5-nucleotidase (NT5E, also known as CD73) are critical mediators among these regulators [56]. CD73 converts AMP to adenosine with phosphate, whereas CD39 can hydrolyze nucleoside-5-triphosphates into nucleoside-5-monophosphate and its products (Table 1). CD39 and CD73 play essential roles in maintaining nucleotide metabolism, while they regulate immune responses via substrate levels of extracellular ATP and adenosine with tumor-promoting and tumor-suppressing effects [57]. Ribose-5-monophosphate and deoxyribose-5-monophosphate are further catalyzed to compose nucleosides and deoxynucleosides mediated by CD73 and CD39 [58]. Furthermore, purine nucleoside phosphorylase (PNP), a ubiquitously expressed homotrimer, catalyzes the reversible phosphorolysis of nucleosides to generate the corresponding purine and pyrimidine base and ribose 1-phosphate, which are converted into purines and pyrimidines [59].

In addition to the de novo biosynthesis pathway, the salvage pathway, which uses free bases that are derived endogenously from the turnover of nucleic acids or exogenously from dietary intake, can generate purines and pyrimidines [60]. The relative importance of salvage versus de novo synthesis likely depends on the growth conditions and on the specific tissue. As the exact steps involved in recycling are only known for purine bases, the final products of the salvage pathway of purines are AMP, IMP, and GMP [59, 60]. During the salvage pathway, ubiquitous PNPs play a key role in catalyzing hypoxanthine–guanine phosphoribosyltransferase (HGPRT) to synthesize the monophosphates (MPs) of inosine (Ino) and guanosine (Guo) [61]. Ribo- and deoxyribonucleosides are converted to the PNP pathway to form only ribonucleotides mediated by adenosine deaminase (ADA) [61]. Uridine–cytidine kinases (UCK1 and UCK2), rate-limiting enzymes involved in the salvage pathway of pyrimidine-nucleotide biosynthesis, convert uridine and cytidine to their corresponding MPs [62].

Nucleotide degradation is another important step in maintaining the homeostasis of nucleotides. Purine nucleotides undergo degradation processes in which nucleotides are converted into nucleosides with the catalysis of nucleotidase in the first step. Adenosine initiates deamination and is catalyzed to Ino and Guo, which are further converted to hypoxanthine and guanine [63]. In the last two steps, hypoxanthine is degraded into uric acid mediated by xanthine dehydrogenase (XDH), and uric acid is then excreted from the body [64, 65]. For pyrimidine catabolism, some pyrimidine molecules (e.g., TMP and dUMP) are sequentially dephosphorylated to their respective bases and converted into open chain amino acids [8]. Dihydropyrimidine dehydrogenase (DPD), a rate-limiting enzyme encoded by *DPYD*, not only initiates the pyrimidine catabolic pathway but also is involved in fluorouracil (5-FU) catabolism [8, 66, 67]. Uridine and thymidine are cleaved and metabolized via amino acids to NH_3_ and CO_2_ mediated by uridine phosphorylase (UPP1) and thymidine phosphorylases (TYMP), respectively [47].

### Key modulators of nucleotide metabolism

Several metabolic enzymes involved in nucleotide metabolism regulate the pathway at mainly the enzyme level. However, the regulation of nucleotide biosynthesis is also controlled by negative feedback of substrate levels such as Pi, purine and pyrimidine analogs [68]. Purine biosynthesis is inhibited by AMP, GMP and Pi, which act on PRPP synthetase, and by adenosine and Guo mono, di or triphosphates (AXP and GXP) at two sites on the PRPP amidotransferase [14, 15]. A key metabolic enzyme involved in pyrimidine biosynthesis, CAD, is controlled by negative feedback of UTP binding to the CPSII domain of CAD and activated by PRPP [9, 69]. CTP synthase catalyzes the transfer of amide nitrogen from glutamine metabolism to UTP to form CTP [70]. Therefore, the activity of CTP synthase regulates the UTP and CTP pools and coordinates the production of pyrimidine and purine nucleotides.

Nucleotide metabolism is not only regulated by metabolic enzymes but also limited and controlled by nucleotide substrates or nucleotide metabolites. Previous studies have observed that acquiring nucleotide bases might be a metabolic bottleneck for cancer development and progression [8, 53, 71]. This provides the rationale for targeting nucleotide metabolism for cancer treatment therapies. Much effort has been made to investigate and explore cancer treatment by disrupting nucleotide metabolism. To date, many chemotherapeutics targeting nucleotide metabolism have been developed and approved for cancer treatments. Herein, it is necessary to investigate and explore the relationship between nucleotide metabolism and cancer, which might provide insights into cancer treatments.

### Nucleotide metabolism and cancer

Multiple metabolic processes are altered in tumorigenesis and cancer progression [68, 72, 73]. The increased demand for nitrogen is regarded as one of the important metabolic hallmarks of cancer cells reported by Pavlova and Thompson [74]. Due to the biological capability of sustaining proliferative signaling in cancers, proliferating cells must synthesize essential nitrogen-containing molecules such as nucleotides [74, 75]. Nucleotide metabolism is considered the most critical link in tumorigenesis and cancer cell replication [76]. One reasonable explanation is that the TME cannot provide sufficient quantities or proportions of nucleotides unless proliferating cells upregulate integrated metabolism of nonessential amino acids, ribose, and one-carbon donors to synthesize these complex molecules [53]. Another potential mechanism is that cancer cells can utilize dysregulated nucleotide metabolism to enhance proliferation and progression [77]. For example, the catalytic activity of DPYD was essential for epithelial-mesenchymal transition (EMT) and cancer progression [78]. Moreover, for the process of nucleotide degradation, downregulated of XDH would contribute to the development and progression of hepatocellular carcinoma, breast cancer, and gastric cancer [79–81].

In diverse cancers, nucleotide metabolism is enriched to meet the demand of uncontrolled and rapid self-proliferation [74]. Meanwhile, upregulated nucleotide metabolism can lead to genomic instability and further carcinogenesis [82]. Several well-known oncogenes and tumor suppressor genes can regulate nucleotide metabolism by signaling pathways to influence tumor growth and progression [74, 83]. A well-known oncogene, *C-myc*, orchestrates nucleotide biosynthesis by upregulating the expression of numerous metabolic enzymes in nucleotide metabolism, such as CAD, TS, and IMPDH [84–86]. Wang et al. [87] indicated that CAD was upregulated in various cancers, including breast cancer, liver cancer, colon cancer with poor clinical outcomes. Furthermore, enriched expression of DHODH and other enzymes of the pyrimidine nucleotide production was found in the MYC-amplified neuroblastoma [88]. MYC is activated by proto-oncogene K-RAS and induces increased transcription of one raw material of nucleotide metabolism, ribose 5’-phosphate isomerase A (RPIA) [89]. It was found that IMPDH-dependent GTP synthesis was linked to MYC’s gene expression programs and suppression of ribosome biogenesis in small-cell lung cancer [90]. Furthermore, mutation of the well-known tumor suppressor gene p53 has been demonstrated to drive tumorigenesis and metastasis [91]. Reddy et al. [92] found that a nucleotide biosynthetic enzyme, guanosine 5'-monophosphate synthase (GMPS), is required for ubiquitin-specific protease 7 (USP7)-mediated stabilization of p53. Mutant p53 alleles can facilitate the expression of nucleotide enzymes such as IMPDH and GMPS [93]. Loss of p53 can activate mTOR complex 1 (mTORC1) to promote de novo pyrimidine and purine synthesis through activation of the CAD enzyme and induction of one-carbon metabolism [36, 94]. In addition, the transcription factor ATF3 can maintain the biosynthesis of purines and pyrimidines and inhibit differentiation in acute myeloid leukemia (AML) [95].

Hence, downregulating nucleotide metabolism could be an effective strategy to kill cancer cells or promote efficacy of cancer treatment. Zhou et al. indicated that inhibiting CDC-like kinase 3 (CLK3), a kinase regulated by *C-myc*, blocks the progression of cholangiocarcinoma through reprogramming nucleotide metabolism [96]. Blocking U2AF homology motif kinase 1 (UHMK1) could inhibit gastric cancer progression by downregulating the expression of purine metabolism-associated target genes [97]. Furthermore, deoxyuridine 5'-triphosphate nucleotidohydrolase inhibition could sensitize TNBC cell lines to fluoropyrimidines and anthracyclines through imbalanced nucleotide pools and increased DNA damage to improve efficacy of these chemotherapeutics [98]. Besides, DNA methyltransferase (DNMT) inhibitor gemcitabine could have synergic effects with PARPi to inhibit breast and ovarian cancers [99]. Binenbaum found that tumor-associated macrophages (TAMs) could release macrophage-derived exosomes, whereas miR-365 generated immunosuppressive effects [100]. miR-365 from exosomes can inactivate gemcitabine by upregulating the triphospho-nucleotide pool in cancer cells and activating cytidine deaminase [100]. One year later, Halbrook et al. provided another explanation for gemcitabine resistance [6]. They observed that TAM-released deoxycytidine, a pyrimidine metabolite, could hamper the antitumor effects of gemcitabine, which inhibits gemcitabine through molecular competition at the level of drug uptake and metabolism [6].

In addition to chemotherapy, the effectiveness of other cancer therapies was associated with altered nucleotide metabolism. As for target therapy, inhibition of DNPH1, a protein that eliminates cytotoxic nucleotide 5-hydroxymethyl-deoxyuridine (hmdU) monophosphate, can resensitize patients with resistance to PARP inhibitors [101]. Also, the *lincNMR* was found to be the first lncRNA to regulate nucleotide metabolism in cancer cells via maintaining activities of key enzymes essential for dNTP biosynthesis [102]. Knockdown of this lncRNA could induce decrease in cell proliferation, senescence, and colony formation [102]. Radiotherapy could decrease metabolites of nucleotide metabolism [103]. Glutamine synthetase and mucin1 were found to promote radiation resistance via facilitating nucleotide biosynthesis in cancer treatment [104, 105].

### Chemotherapeutic agents disrupt nucleotide metabolism to suppress cancers

As discussed above, nucleotide metabolism plays a crucial role in carcinogenesis and cancer progression. Much effort has been devoted to cancer treatment by targeting nucleotide metabolism [1, 13, 106]. Drugs such as 5-FU and gemcitabine block nucleotide metabolism and are an important part of chemotherapy [107, 108]. To date, chemotherapy is the keystone treatment in the adjuvant setting in many types of cancer [109, 110]. There are over 20 approved nucleotide and nucleotide analogs used in cancer chemotherapies, which account for nearly 20% of all drugs in cancer treatment (Table 2). Therapeutic agents targeting nucleotide metabolism can be classified into three primary categories, including purine analogs, pyrimidine analogs, and metabolic enzymatic inhibitors, based on their structures and mechanisms [107, 111].

**Table 2:** Clinically FDA-approved drugs targeting nucleotide metabolism in cancer

Therapeutic agents	Inhibition targets/target pathways	Approved indication	First approved date	References
Mercaptopurine	Hypoxanthine–guanine phosphoribosyltransferase, amidophosphoribosyltransferase, Inosine-5'-monophosphate dehydrogenase	Acute lymphatic leukemia	1953	[112, 113]
Methotrexate	Dihydrofolate reductase, thymidylate synthase, aminoimidazole carboxamide ribonucleotide transformylase, and amido phosphoribosyltransferase	Acute lymphoblastic leukemia, gestational choriocarcinoma, chorioadenoma destruens, hydatidiform mole, breast cancer, epidermoid cancer of the head and neck, advanced mycosis fungoides, lung cancer, and advanced non-Hodgkin’s lymphoma	1953	[114]
Fluorouracil	Thymidylate synthase	Colon, esophageal, gastric, rectum, breast, biliary tract, stomach, head and neck, cervical, pancreas and renal cell cancer	1962	[108]
Thioguanine	DNA	Acute non-lymphocytic leukemias	1966	[113, 115]
Cytarabine	DNA polymerase	Acute non-lymphocytic leukemia	1969	[116]
Floxuridine	Thymidylate synthase	Gastrointestinal adenocarcinoma, liver cancer	1970	[117, 118]
Cisplatin	DNA	Testicular tumors, ovarian tumors and bladder cancer	1978	[119]
Carboplatin	DNA	Advanced ovarian carcinoma	1989	[120]
Fludarabine	Ribonucleoside-diphosphate reductase large subunit, DNA polymerase alpha catalytic subunit	Chronic lymphocytic leukemia	1991	[121, 122]
Cladribine	Ribonucleoside-diphosphate reductase, DNA polymerase	Active hairy cell leukemia, chronic lymphocytic leukemia, non-Hodgkin's lymphoma	1991	[123]
Pentostatin	Adenosine deaminase	Hairy cell leukemia	1991	[29]
Hydroxyurea	Ribonucleotide reductase	Acute myeloid leukemia	1995	[124]
Gemcitabine	Ribonucleoside-diphosphate reductase, thymidylate synthase, UMP-CMP kinase	Ovarian, lung, breast and pancreas cancer	1996	[125, 126]
Dacarbazine	Unspecific, DNA synthesis	Malignant melanoma, Hodgkin’s disease	1998	[127]
Capecitabine	Thymidylate synthase	Breast and colon cancer	1998	[128]
Clofarabine	Ribonucleoside-diphosphate reductase, DNA polymerase	Acute lymphocytic leukemia	2004	[129, 130]
Azacytidine	Cysteine and methionine metabolism	Chronic myelomonocytic leukemia	2004	[131, 132]
Nelarabine	Ara-G triphosphate	Acute T-cell lymphoblastic leukemia, T-cell lymphoblastic lymphoma	2005	[133]
Decitabine	DNA (cytosine-5)-methyltransferase 3A and 3B	Myelodysplastic syndromes (MDS)	2006	[134]
Oxaliplatin	DNA	Colorectal cancer	2009	[120]
Idelalisib	Phosphatidylinositol 4,5-bisphosphate 3-kinase catalytic subunit	Chronic lymphocytic leukemia, follicular B-cell non-Hodgkin lymphoma, and small lymphocytic lymphoma	2014	[135, 136]
Tipiracil	Thymidine phosphorylase	Colorectal cancer	2015	[137]
Pemetrexed	Thymidylate synthase, Bifunctional purine biosynthesis protein PURH, Dihydrofolate reductase, Trifunctional purine biosynthetic protein adenosine-3	Mesothelioma, NSCLC	2015	[138]

*NSCLC* non-small-cell lung cancer

**Fig. 1:**
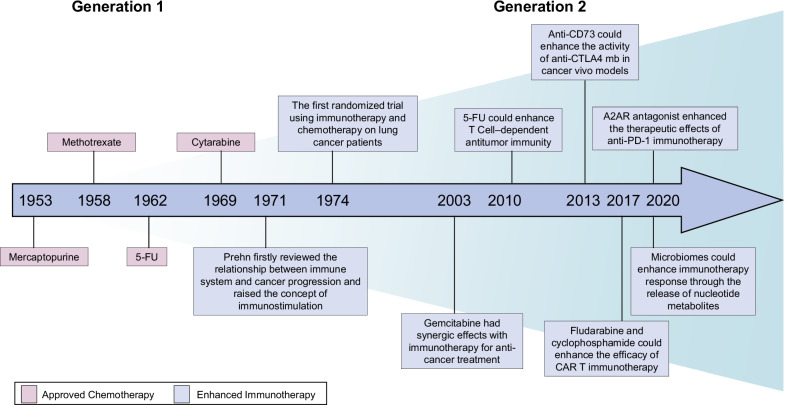
Historical development and breakthroughs in targeting nucleotide metabolism in cancer treatment. Targeting nucleotide metabolism in cancer treatment could be divided into two generations. In the Generation 1, targeting nucleotide metabolism was designed as chemotherapeutics to treat cancer. In the Generation 2, immunotherapy could enhance its efficacy with therapeutic agents blocking nucleotide metabolism

Purine analog antimetabolites include thiopurines, deoxypurines, arabinose purine analogs, and base-modified purine nucleosides [107]. Although thiopurines were introduced into the clinic in the early era of cancer chemotherapy, few representative drugs are known. In the early 1950s, Elion’s group discovered that 6-mercaptopurine and thioguanine, two of the earliest thiopurine analogs found, could hamper the growth of Lactobacillus casei [134]. In 1953, 6-mercaptopurine was proven to have effects in clinical trials by inhibiting the phosphorylation and hydrolysis of nucleosides and was approved by the FDA for the treatment of childhood leukemia (Fig. 1) [111–113]. Subsequently, thioguanine received approval for acute non-lymphocytic leukemias in 1966 [113, 115]. In addition, thiopurines have various clinical applications outside hematologic cancers, such as autoimmune diseases and organ transplantation rejection [139, 140]. Deoxyadenosine analogs have been found to be resistant to adenosine deaminase activity and may be phosphorylated into their triphosphate forms in the cell [141]. Cladribine, an approved deoxyadenosine analog, was used as a first-line monotherapy for hairy cell leukemia [123]. Clofarabine, a second-generation deoxyadenosine analog with more stability than first-generation drugs such as cladribine, was indicated for relapsed or refractory pediatric acute lymphoblastic leukemias [129, 130, 142]. Arabinose purine analogs consist of nelarabine and fludarabine. They were approved for the treatment of relapsed T cell acute lymphocytic leukemia, relapsed T cell lymphoblastic lymphoma, and chronic lymphocytic leukemia [121, 122, 133]. Base-modified purine nucleosides, including 8-chloro-adenosine, tocladesine, and forodesine, have not yet received approval from the FDA.

Pyrimidine analogs include fluorinated pyrimidines, azanucleosides, ribosugar-modified cytidine analogs, cytarabine, and its prodrugs [107]. Fluorinated pyrimidines contain 5-FU, capecitabine, floxuridine, and tipiracil hydrochloride (TAS-102) [107]. In 1954, Rutman et al. [68] observed that exogenous uracil is utilized for nucleic acid formation during the process of hepatic carcinogenesis in vivo. Based on this finding and previous understanding of thymidylate synthase, Heidelberger and colleagues synthesized fluorouracil [143]. Then, 5-FU received FDA approval in 1960 [144–146], and it currently has further extensive indications for various malignancies, such as gastrointestinal cancer, breast cancer, and renal cell cancer [108, 147]. In 1990, Hertel et al. [148] synthesized gemcitabine, a novel pyrimidine antimetabolite, and discovered its excellent antitumor activity in experimental tumor models. Gemcitabine was originally tested in hematological malignancies and found to have outstanding antitumor effects not only in hematological cancers but also in other solid tumors [107]. Currently, it has been approved by the FDA for ovarian, lung, breast and pancreatic cancer (Table 2). Floxuridine is converted into floxuridine-5’-monophosphate II mediated by thymidine kinase; thus, using floxuridine induces inhibition of thymidylate synthase, and it gained approval for metastatic colon and colorectal cancers from the FDA in 1970 [149]. TAS-102 refers to the combination of tipiracil hydrochloride and trifluorothymidine and was approved for colorectal cancer patients [150, 151]. In the category of azanucleosides, decitabine and azacytidine inhibit DNA methylation to achieve antitumor effects [131, 132, 134]. They received approval for myelodysplastic syndrome, known as preleukemia [152]. Gemcitabine, a representative drug of ribosugar-modified cytidine analogs, disrupts DNA biosynthesis through cell cycle arrest induced by “masked chain termination” [153]. Cytarabine was observed to have expected antitumor effects in vivo in the 1960s, tested rapidly in animal models and clinical trials, and subsequently received approval from the FDA [154].

Specific inhibitors target metabolic enzymes of nucleotide metabolism, providing a secondary mode of action that inhibits cell growth. However, most specific enzymatic blockers have not gained FDA approval and remain in phase I/II clinical trials. Enzymatic blockers of nucleotide metabolism can be further divided into purine, pyrimidine and general inhibitors. For purine enzymatic blockers, IMDPH has specific inhibitors, such as mizoribine, merimepodib and mycophenolic mofetil, which have not been approved for cancer therapy [36–40, 155]. A phase I clinical trial testing mycophenolic mofetil on pancreatic cancer has been completed (NCT00997958). A deoxyadenosine agent, pentostatin, targets ADA and gained FDA approval for hairy cell leukemia [29]. Pemetrexed, an antifolate, inhibits folate-dependent enzymes involved in the de novo biosynthesis of thymidine and purine nucleotides such as TS and GART. MLN4924, a structural analog of AMP, inhibits carcinogenesis by blocking the proteasomal degradation pathway [156]. It has been tested and proven to be safe and effective in many clinical trials on various cancers (e.g., melanoma, acute myeloid leukemia, and lymphoma) [157]. In contrast, there are more specific inhibitors of enzymes involved in pyrimidine nucleotide metabolism. As a key rate-limiting enzyme, DHODH is a target of several drugs, such as teriflunomide and leflunomide [158]. Although these two therapeutic agents have been reported to achieve antiproliferative effects on cancers such as multiple myeloma, NSCLC, and neuroblastomas, none to date has gained FDA approval for cancer [158, 159]. Another drug, gimeracil, has been shown to play an antineoplastic role by blocking DPYD to prevent the breakdown of 5-FU [48]. Furthermore, there are several inhibitors, such as CD73 and CD39, that target the common pathway of both purine and pyrimidine metabolism [160]. Anti-CD73 inhibitors include oleclumab, AB680, and adenosine 5'-(alpha, beta-methylene) diphosphate (APCP) [21, 58]. Oleclumab has shown safety and efficacy in combination with durvalumab in pancreatic cancers [161]. Oleclumab and other anti-CD73 monoclonal antibodies (mAbs) are currently being investigated in phase I/II clinical trials [21]. Similarly, pharmacological CD39 inhibitors, including sodium polyoxotungstate (POM-1), antisense oligonucleotides (ASOs), and TTX-030, have been evaluated as monotherapies and in combination with chemotherapy and/or immunotherapy in currently undergoing clinical trials (Table 3) [162]. Due to the biological and clinical significance of these two molecules, dual blockers have indicated a potential synergistic antitumor effect in several preclinical studies [22].

**Table 3:** Cancer immunotherapy with additional nucleotide-metabolic targets versus immunotherapy monotherapy in the clinical trials

Therapeutic agents	Therapeutic targets	Accompanied immunotherapy	Participants	ClinicalTrials.gov identifier	Phase	Status
Oleclumab	CD73	Durvalumab	Triple negative breast cancer	NCT03616886	Phase I/II	Recruiting
ASOs	CD39	Durvalumab	Diffuse Large B-cell Lymphoma	NCT02549651	Phase I	Completed
Gemcitabine	Ribonucleoside-diphosphate reductase, thymidylate synthase, UMP-CMP kinase	Tislelizumab	Urothelial carcinoma	NCT04570410	Phase II	Recruiting
		Durvalumab	Advanced solid tumors	NCT03907475	Phase II	Recruiting
Pemetrexed	Thymidylate synthase, bifunctional purine biosynthesis	Pembrolizumab	NSCLC	NCT04533451	Phase II	Recruiting
		Pembrolizumab	NSCLC	NCT04547504	Phase III	Recruiting
Gemcitabine and 5-FU	Thymidylate synthase	Cabiralizumab and nivolumab	Advanced pancreatic cancer	NCT03336216	Phase II	Active, not recruiting
Pemetrexed/ Gemcitabine	Thymidylate synthase	Sintilimab	NSCLC	NCT04728724	Phase II	Not yet recruiting
Capecitabine	Thymidylate synthase	Zanidatamab	Her2-expressing cancers	NCT02892123	Phase I	Recruiting
		Interferon and interleukin	Advanced renal cell carcinoma	NCT00311467	Phase III	Terminated
Azacitidine	Cysteine and methionine metabolism	Pembrolizumab	Metastatic cancer	NCT02959437	Phase I/II	Completed
		Anti-OX40 antibody	AML	NCT03390296	Phase I/II	Recruiting
Cytarabine	DNA polymerase	Gemtuzumab	AML	NCT00006265	Phase II	Completed
		NY-ESO-1T Cells	Synovial Sarcoma	NCT01343043	Phase I	Completed
		Autologous HER2-specific T cells	Advanced sarcoma	NCT00902044	Phase I	Active, not recruiting
		Personalized neoantigen adoptive cell therapy	Solid tumors	NCT04596033	Phase I	Recruiting
		DNR.NPC-specific T cells	Nasopharyngeal carcinoma	NCT02065362	Phase I	Active, not recruiting
Dacarbazine	DNA synthesis	CPG 7909	Melanoma	NCT00070642	Phase II	Completed
		Melan-A	Melanoma	NCT00559026	Phase I	Completed
Decitabine	DNA (cytosine-5)-methyltransferase 3A and 3B	Nivolumab	NSCLC	NCT02664181	Phase II	Active, not recruiting
Idelalisib	Phosphatidylinositol 4,5-bisphosphate 3-kinase catalytic subunit	Pembrolizumab	CLL and non-Hodgkin lymphoma	NCT02332980	Phase II	Recruiting

*CD73* ecto-5-nucleotidase, *CD39* ecto-nucleoside triphosphate diphosphohydrolase-1, *NSCLC* non-small-cell lung cancer, *AML* acute myeloid leukemia, *CLL* chronic lymphocytic leukemia

Based on the summary of therapeutic agents targeting nucleotide metabolism, this study demonstrates the significance and reliability of the general effectiveness and safety of targeting nucleotide metabolism as chemotherapy in cancers. These research results have not only greatly enriched the understanding of the regulatory mechanism of nucleotide metabolism in cancers but also provided insights into the clinical development of new specific therapeutic drugs. Although chemotherapy is still the keystone of all cancer treatment therapies to date in the adjuvant setting, the effectiveness of chemotherapy is hampered by drug resistance and adverse side effects [163, 164]. Resistance to chemotherapeutics is caused by several mechanisms, including gene mutations, chromosomal instability, and DNA repair [164]. These disadvantages of cytotoxic chemotherapy have forced scientists and clinicians to consider other systemic treatment therapies.

With continuous in-depth study of chemotherapy and cancers, our understanding of nucleotide metabolism in tumors has revealed their non-proliferative effects beyond their effects on cancer cell proliferation [8]. Dysregulated nucleotide metabolism has been found to alter the immune microenvironment and affect the host immune response [72, 165]. Altered immune components in the TME indicate the potential application of immunotherapy, which is considered one of most promising approaches to precisely killing tumors and maintaining the immune microenvironment in the era of precision medicine. Therefore, it is important to explore the interactions between nucleotide metabolism and cancer immunity to provide a theoretical basis for cancer treatments outside chemotherapy.

### Interaction between nucleotide metabolism and cancer immunity

Nucleotide metabolism provides genetic materials and energy resources for immune system activation and proliferation. Dysregulated nucleotide metabolism induces inhibition or activation of the immune response [68]. Endogenous and exogenous nucleotides and their metabolites from host and microbial infection activate the immune system through several host receptors, such as Toll-like receptors (TLRs), RIG-like receptors (RLRs), NOD-like receptors (NLRs), purinergic receptors, and adenosine receptors [56, 166–168].

**Fig. 2:**
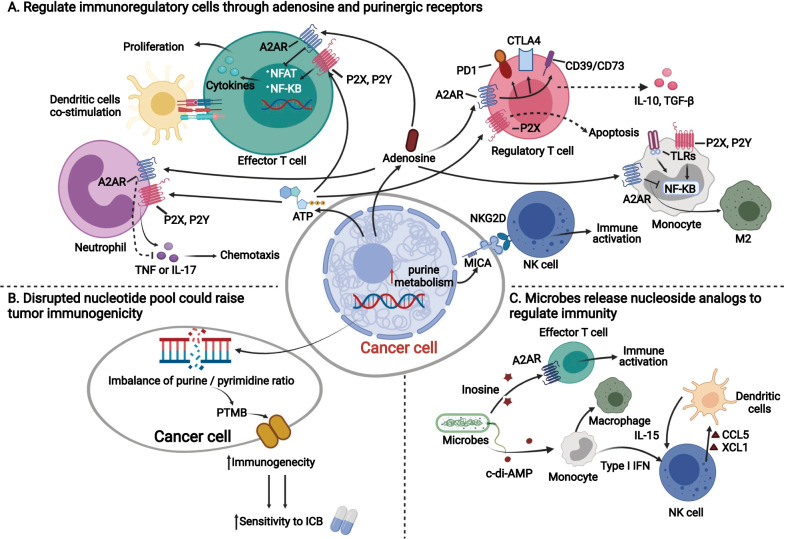
Interactions between nucleotide metabolism and host immunity. Cancer cells could release metabolites from nucleotide metabolism, such as ATP, adenosine to **A** Regulate immunoregulatory cells through adenosine and purinergic receptors; **B** In cancer cells, disrupted nucleotide pool could raise tumor immunogenicity; **C** Microbes release nucleoside analogs to regulate immunity. *A2AR* adenosine 2A receptor, *P2X* purinergic P2X receptor, *P2Y* purinergic P2Y receptor, *TLRs* Toll-like receptors, *CD39* ecto-nucleoside triphosphate diphosphohydrolase-1, *CD73* ecto-5-nucleotidase, *M2* M2-type macrophage, *MICA* major histocompatibility complex class I-related chain A, *NFAT* nuclear factor of activated T cells, *PTMB* pyrimidine-rich transversion mutational bias, *TAM* tumor-associated macrophage

Purine analogs, such as released extracellular ATP or adenosine, activate purinergic receptors and adenosine receptors from immune cells to promote or inhibit the immune response [68, 169] (Fig. 2A). Adenosine acts on several adenosine receptors, including A1R, A2AR, A2BR, and A3R, and mediates its regulatory roles between nucleotide metabolism and the immune response. These four receptors have different affinities toward adenosine levels. A1R, A2AR and A3R more easily connect with accumulated adenosine, while A2BR can respond only when it meets high concentrations of adenosine in some pathological conditions [170]. Although A2BR can only be activated with relatively high concentrations of adenosine, blockade of A2BR can contribute to inhibiting the growth of tumors in vivo [171, 172]. Extracellular adenosines can act on A2AR in Treg cells and effector T cells, causing the activation of CD39, CD73, programmed cell death protein 1 (PD-1), and cytotoxic T lymphocyte antigen 4 (CTLA-4) on Treg cells and inhibiting the secretion of IL-2 and other cytokines [173, 174]. Specifically, the inhibitory mechanism of adenosine is mainly via inhibition of Ca^2+^ influx and nuclear factor of activated T cells (NFAT) stimulation [175]. Hence, adenosine inhibits effector T lymphocyte proliferation and the secretion of inflammatory cytokines and is therefore critical for both innate and adaptive immune responses [169]. In contrast, high levels of ATP can activate P2X on Treg cells to drive apoptosis and bind to P2X and P2Y receptors on effector T cells to facilitate their proliferation [173, 174]. Moreover, cancer-derived purine metabolites carried by exosomes work as potential contributors to tumor immune escape [176].

Furthermore, accumulated adenosine and ATP cause extensive immune inhibition and activation in similar manners on other components in the TME, such as natural killer (NK) cells, monocytes and neutrophils. ATP activates NK cells by P2 receptors and promotes proliferation and NK-mediated innate immunity, while CD73-derived adenosine stimulates A2AR to suppress antitumor immunity [177, 178]. Monocytes can polarize into macrophages with two different immunostimulatory characteristics. Extracellular ATP can act on TLRs, P2X, and P2Y of monocytes, subsequently inducing the polarization of M1 macrophages via NF-κB [169]. However, high levels of adenosine can block NF-κB, which promotes monocytes to polarize into M2 macrophages [169] (Fig. 2A). In neutrophils, adenosine stimulates A2AR via activation of the NF-kB pathway, limiting NK cell activation and IFNγ production but increasing TGF-β and IL-10 secretion [179]. Nils Ludwig et al. [176] found that head and neck squamous cell carcinoma (HNSCC) cells secrete purine metabolites in exosomes, whereas immunosuppressive adenosine and Ino are predominant. The amounts of shuttled purine metabolites in exosomes are significantly reduced with increased cancer stages and progression [176]. CD39 and CD73 are primarily involved in hydrolyzing proinflammatory ATP to generate immunosuppressive adenosine to regulate the host immune system [180]. In addition to mediating the rate-limiting step for conversion of extracellular ATP and adenosine, CD39 and CD73 promote angiogenesis of endothelial cells and lymphocyte adhesion to the endothelium, resulting in an increased risk of metastatic progression [181, 182]. In addition, oxidative stress controls Treg cell apoptosis, wherein it promotes the release and conversion of ATP to adenosine via CD39 and CD73 and mediates immunosuppression via the adenosine and A2A pathways [183]. In addition, cross talk between immune cells and cancer cells is mediated by products of nucleotide metabolism to some extent. Upregulated purine metabolism in cancer cells can induce increased expression of MICA, binding natural killer group 2D receptor (NKG2D) expressed in NK cells and inducing proliferation of NK cells and immune response [184].

Compared to purine and its analogs, there have been relatively fewer studies on the relationship between pyrimidine metabolism and immunity. A recent study had indicated that supplement of uridine diphosphate (UDP) could activate immune responses in vivo [185]. Lee et al. found that urea cycle dysregulation could enhance pyrimidine synthesis via changes in nitrogen metabolism and activation of CAD [186]. Excessive pyrimidines cause an increased pyrimidine/purine ratio and purine-to-pyrimidine transversion mutations (PTMB), which are associated with enhanced immunogenicity and a better response to immune checkpoint inhibitors [186]. Similarly, Keshet also indicated consistent findings that the IMDPH inhibitor mizoribine could block purine biosynthesis in ASS1-expressing tumors, disrupt the balance of nucleotide pools, release many immunoproteasomes, and subsequently generate PTMB [187]. Therefore, a dysregulated nucleotide pool raising tumor immunogenicity is another important mechanism between cancer nucleotide metabolism and cancer immunity (Fig. 2B).

Recently, the linkage between microbes and cancer immunity has raised interest among the scientific community, while nucleotide analogs play an important role in this linkage [5, 188–190] (Fig. 2C). Several microbes could act on the adenosine pathway to affect cancer immunity and enhance the efficacy of immune checkpoint blockage (ICB) therapies in animal models [5]. Specifically, *Bifidobacterium pseudolongum*, *Lactobacillus johnsonii*, and Olsenella species were found to promote ICB therapies by raising CD4^+^ and CD8^+^ T cell activation [5]. Among these three microbes, *B. pseudolongum* promoted the immune response by producing the metabolite Ino and directly acting on A2AR expressed in naïve T cells. Microbe-derived Ino could independently activate A2AR and upregulate the cAMP-PKA pathway to initiate Th1 differentiation and costimulate dendritic cells [5]. In addition, several favorable microbes can secrete and release c-di-AMP to induce the activation of monocytes [189], which activates the proliferation and secretion of adaptive immune cells such as NK cells and DC cells through the STING pathway [189]. Hence, based on this rationale, fecal microbiota transplantation from patients who are sensitive to ICB therapies could help to improve the efficacy of immunotherapy [189].

Indeed, targeting nucleotide metabolism can directly alleviate immune suppression; for example, secreted purines can directly bind inhibitory receptors on immune cells [191]. However, targeting nucleotide biosynthesis can inhibit the rapid proliferation of not only cancer cells but also immune cells [192]. Hence, this strategy exerts secondary effects to block the host immune response, as adaptive immunity depends on rapid proliferation of lymphocytes [192]. Therefore, integrated effects must be considered when developing therapeutic strategies that target nucleotide metabolism [165].

These findings highlight the solid association between nucleotide metabolism and antitumor immunity. Here, we elaborate the idea that antimetabolites or specific inhibitors targeting nucleotide metabolism promote infiltration of the immune microenvironment and facilitate the efficacy of immunotherapy, such as anti-PD-1 or CAR T cell treatment.

### Targeting nucleotide metabolism could enhance the efficacy of cancer immunotherapy in experimental and clinical studies

As we mentioned above, there are close interactions between nucleotide metabolism and immunity, which provides rational potential for immunotherapy with antinucleotide metabolism agents in cancer patients. Immunotherapy, as a novel cancer treatment, has captured considerable attention across the oncology community in the past decade. It has made huge progress in several kinds of malignancies, such as non-small-cell lung cancer (NSCLC) [193], melanoma [194], lymphoma [195], and metastatic bladder cancer [196]. However, in some cold tumors, such as triple-negative breast cancer (TNBC), immune checkpoint blockade monotherapy does not seem to achieve our expectations [197]. Improving the efficacy of cancer immunotherapy in the clinic is an urgent problem for oncologists. To solve this conundrum, scientists have attempted to combine immunotherapy with other adjuvant treatment therapies, such as chemotherapy, radiotherapy, and targeted therapy, and have observed clinical benefits in various malignancies [198–202]. However, although olaparib accompanied with durvalumab could improve pathological completed rates of HER2-negative breast cancer patients in I-SPY 2 trial and radiotherapy was found to increase responses combined with immunotherapy in metastatic NSCLC, chemotherapy was still the major partner of immunotherapy [203–205].

Chemotherapy was considered immunosuppressive, causing neutropenia and lymphopenia and other adverse side effects [206]. However, Prehn first reviewed the relationship between the immune system and cancer progression and raised the concept of immunostimulation in 1971, which indicated the accelerated growth of tumors stimulated by immune factors [207]. Macpherson et al. further discussed the role of immunostimulation in immunochemotherapy and explained why combined immunotherapy and chemotherapy had synergistic effects on cancer treatment [208]. Immunotherapy promotes more active tumor cells into the cell cycle, while chemotherapy inhibiting the synthesis and integrity of nucleotides could achieve more profound antitumor effects [208]. Half a century ago, Stewart et al. [209] performed the first randomized trial of a combination of immunotherapy and chemotherapy in lung cancer patients. They observed that the third experimental group receiving methotrexate and immunization had longer disease-free survival than groups receiving chemotherapy monotherapy or single immunotherapy [209]. Another early randomized trial compared chemotherapy, immunotherapy, and immunochemotherapy in melanoma patients but failed to achieve the expected results [210].

In 2003, Nowak et al. [206, 211] made an important discovery that additional agents targeting nucleotide metabolism could have synergic effects in the combination of immunotherapy for cancer treatment (Fig. 1). They found that gemcitabine could raise CD4 and CD8 T-cell infiltration and subsequently promote antigen-specific cellular antitumor immunity [206, 211]. Since chemotherapy has generally been considered immunosuppressive in past decades, these findings provide insights into the synergy between cytotoxic chemotherapy and immunotherapy. Therefore, what induced synergism of immunochemotherapy could not be simply explained by blockade of more cancer cells into the S phase of the cell cycle. The chemotherapeutic agents mentioned above mainly targeted the different sections of nucleotide metabolism. Targeting nucleotide metabolism could drive the activation of adaptive immune responses, which could facilitate antitumor effects accompanied by immunotherapy. Taken together, nucleotide metabolism could be a promising target to improve the effectiveness of cancer immunotherapy.

**Fig. 3:**
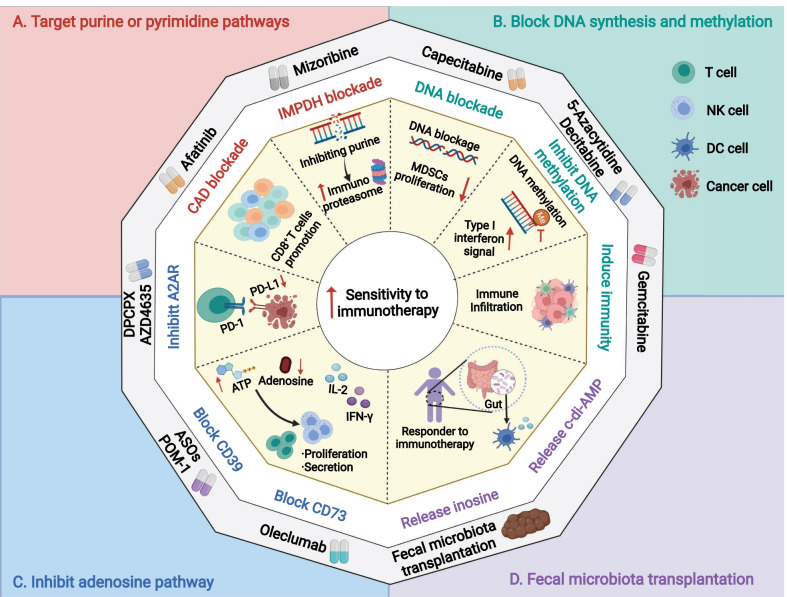
Therapeutic strategies to exploit the nucleotide metabolism–immunity interplay in the clinic. **A** Targeting purine or pyrimidine pathways; **B** blocking DNA synthesis; **C** inhibiting adenosine pathway; **D** fecal microbiota transplantation. *CAD* carbamoyl phosphate synthetase, aspartyl transcarbamoylase, and dihydroorotase, *IMPDH* inosine monophosphate dehydrogenase, *MDSCs* myeloid-derived suppressive cells, *A2AR* adenosine 2A receptor, *CD39* ecto-nucleoside triphosphate diphosphohydrolase-1, *ASOs* antisense oligonucleotides, *CD73* ecto-5-nucleotidase

To illustrate the recent progress on therapeutic agents targeting nucleotide metabolism in cancer treatment, we classify them into four categories based on the nucleotide metabolism pathway as follows: (A) targeting purine and pyrimidine pathways, (B) blocking DNA synthesis, (C) inhibiting the adenosine pathway, and (D) fecal microbiota transplantation (Fig. 3).

Directly targeting purine and pyrimidine metabolism could be an effective strategy for enhancing cancer immunotherapy (Fig. 3A). As mentioned above, mizoribine could inhibit purine synthesis and promote the release of the immunoproteasome [187]. This could induce an increased response of autologous CD8^+^ T cells to anti-PD1 therapies in specific ASS1-expressing cancers [187]. Furthermore, numerous studies have demonstrated that blocking DHODH could efficiently inhibit the growth of various malignancies, including glioblastoma stem cells [41], acute myeloid leukemia [46], and small-cell lung cancer [212]. However, few studies have investigated whether DHODH inhibitors have synergistic effects with immunotherapy. A DHODH inhibitor, P1788, was identified and found that DHODH inhibition could enhance cellular antitumor immunity via increased interferon signaling [213]. This study provided insights into enhancing innate immunity through blockade of de novo pyrimidine biosynthesis. Another recent study indicated that afatinib, a kind of EGFR-tyrosine kinase inhibitor (TKI), could be a potential agent to enhance the efficacy of ICB therapies by targeting another important rate-limiting enzyme of nucleotide metabolism, CAD [214]. Blocking CAD and nucleotide metabolism in cytotoxic T cells causes immune suppression in the short term, but the proliferation of CD8^+^ T cells unexpectedly rebounds following long-term treatment [214].

Targeting the common pathway of both purine and pyrimidine metabolism is generally considered chemotherapy to arrest tumor cells in the cell cycle. Transformation between ribose-5-monophosphate and ribose-5-triphosphate is an essential step in complete nucleotide biosynthesis. Common chemotherapeutic agents, such as gemcitabine, capecitabine, and 5-FU, have been indicated in numerous malignancies, but recent studies have observed the potential to have synergistic effects with immunotherapy (Fig. 3B). Gemcitabine was found to enhance antitumor immunity via increased antigen cross-presentation, T lymphocyte expansion, and infiltration in solid tumors [206, 211]. Furthermore, scientists observed that gemcitabine and 5-FU could selectively kill myeloid-derived suppressor cells (MDSCs), and other immune components in the TME, such as T cells, B cells, NK cells, or DC cells, remained unchanged [215]. 5-FU could induce IFN-γ production by tumor-specific CD8^+^ T cells infiltrating the tumor and promote T cell-dependent antitumor responses by eliminating MDSCs in vivo [215]. Similarly, capecitabine, an oral prodrug of 5-FU, enhances immunotherapy efficacy in glioblastoma [216]. In a phase 0/I dose-escalation clinical trial, Peereboom et al. [216] proved the effectiveness and tolerance of additional metronomic capecitabine with bevacizumab in glioblastoma patients. The addition of capecitabine increased cytotoxic immune infiltrations by inhibiting MDSCs and facilitated immunotherapy. In addition to these chemotherapeutic agents, DNMT inhibitors contribute to an increase in the activation and cytolytic activity of CD8^+^ T cells [217]. Decitabine upregulates T cell activation and promotes T cell-based immunotherapy in lung cancer in vivo models [218]. Similarly, 5-azacytidine increases CD45^+^ immune cells, CD8^+^ T cells, and NK cells [219]. A combination of DNMT inhibitors plus the immune checkpoint inhibitor anti-PD-1 enhances antitumor effects in ovarian cancer [219]. In addition, a phase I trial confirmed the safety and efficacy of additional fludarabine, and targeting the ribonucleoside-diphosphate reductase large subunit, the DNA polymerase alpha catalytic subunit, increased the efficacy of CAR T cell immunotherapy in patients with neuroblastoma [132].

Adenosine and its analogs have been extensively recognized as key metabolites in modulating the immune microenvironment [57, 220]. Adenosine receptor expression is negatively correlated with immune infiltration and prognosis in various cancers [221–224]. Adenosine receptors are considered perfect targets by several inhibitors, such as DPCPX and AZD4635, to activate immunosuppressive adenosine signaling [220, 225] (Fig. 3C). An A2AR antagonist, AZD4635 could prompt T cell proliferation and interferon gamma production and reduce the tumor load in multiple myeloma (MM) models [220]. In addition, another A2AR inhibitor, DPCPX could upregulate PD-L1 via the transcription factor ATF3 [225]. Authors also proved the potential benefits of the synergism of DPCPX and a PD-1 mAb in NSCLC or melanoma models [225]. Moreover, another A2AR antagonist, CPI-144, could enhance the efficacy of anti-PD-L1 or anti-CTLA-4 treatment in preclinical models [226]. Recently, Giuffrida et al. [227, 228] found that deletion of A2AR by CRISPR/Cas9 or shRNA could promote antitumor immunity with CAR T immunotherapy in vivo. Furthermore, extracellular nucleotides were found to stimulate purinergic receptors to induce chemotaxis and adhesion of lung cancer cells [229]. This evidence highlights the potential applications of agents targeting adenosine–adenosine receptors combined with immunotherapy for cancer treatment.

Given the critical position of CD39 and CD73 in downmodulating effector antitumor immunity through the generation of adenosine, strategies targeting these central mediators could enhance cancer immunotherapy. In human peripheral blood, CD39 and CD73 are extensively expressed on immune subsets, especially in B cells and CD4^+^ T cells [21]. CD39 antagonists hamper the proliferation of CD4^+^ and CD8^+^ T cells via an adenosine-dependent pattern and ATPase activity in vivo [230]. Furthermore, POM-1, a specific inhibitor of CD39, increases IFN-gamma or IL-2 secretion upon anti-CD3/CD28 stimulation and enhances antitumor immunity in vitro [231]. POM-1 also effectively suppresses metastases by activating NK cells when used in combination with immunotherapy, such as anti-PD-1, anti-CTLA-4 and IL-2 [232]. Kashyap and coworkers demonstrated that ASOs lead to improved CD8^+^ T cell proliferation and reduced Treg and tumor-associated macrophages [233] (Fig. 3C).

Regarding CD73, previous studies have indicated that CD73 promotes tumor metastasis by blocking the function of NK cells [234]. Previous studies have observed its prognostic value and found that CD73 expression is conversely associated with prognosis and antitumor immunity in TNBC and renal cell carcinoma [235, 236]. An early phase of a clinical trial proved that oleclumab significantly altered several immune subpopulations in the TIME, including increased CD8^+^ T cells and activated macrophages [237]. Data from single-cell RNA sequencing have also indicated that CD73 is a specific immunotherapeutic target for facilitating ICB therapies in glioblastoma (GBM) [238]. Anti-CD73 enhances the activity of anti-CTLA4 mAbs through activation of the T-cell response in in vivo cancer models such as colon cancer, prostate cancer, melanoma, and glioblastoma [238–240]. A combinational approach of CD39 and CD73 inhibition synergistically enhances antitumor immunity. As immune cells infiltrating the tumor coexpress CD39 and CD73 in association with other coinhibitory molecules (e.g., CTLA4 and PD-L1), dual blockade of both CD39 and CD73 has been proposed with the aim of controlling the immunosuppressive role of adenosine signaling while minimizing the side effects of ICB [22, 220, 241–243].

As we mentioned above, microbes could modulate host immunity through release of different metabolites, such as Ino and c-di-AMP (Fig. 2C). Hence, many preclinical studies had revealed that the potential effectiveness of fecal microbiota transplantation (FMT) from responders to overcome resistance to immunotherapy [244–246]. Based on this rationale, scientist designed and evaluated FMT in clinical trials; they had demonstrated that FMT together with anti-PD-1 could treat refractory melanoma patients [247, 248]. Therefore, FMT could be considered to improve the efficacy of immunotherapy in other solid tumors, which were previously regarded insensitivity to immunotherapy. Extraction of microbe-release nucleotide analogs with immunoregulatory functions such as Ino and c-di-AMP and exogenous supplement of these materials might be helpful in improving immunotherapy, warranting further exploration and validation.

Targeting a specific purine or pyrimidine metabolic pathway can induce a nucleotide pool imbalance by decreasing the biochemistry levels of one pool relative to the other [165]. Imbalance of nucleotide pools between purines and pyrimidines could generate PTMB and subsequently increase neoantigens and immunogenicity as mentioned above [186, 187, 249]. An integrated study of clinical and genomic data found that higher somatic tumor mutational burden (TMB) was correlated with better survival and response to ICB therapies across multiple cancer types [250]. Therefore, it is possible to induce more mutations by disrupting the nucleotide-pool balance to raise the efficacy of immunotherapy during cancer treatment, warranting further exploration and validation.

The above evidence demonstrates that targeting nucleotide metabolism (cytotoxic chemotherapy or specific enzyme inhibitors) can facilitate immunotherapy in numerous cancer types, even in several well-known cold tumors. After exploration of the safety and efficacy of therapeutic agents targeting nucleotide metabolism, clinical trials could be performed, especially in advanced or progressed diseases after conventional first-line therapies.

### Future clinical trials of cancer immunotherapy with additional nucleotide antimetabolites

After a conceptual breakthrough in immunotherapy with nucleotide antimetabolites, some clinical trials testing the safety and efficacy in cancer patients have been initiated or completed. Table 3 summarizes currently undergoing and completed clinical trials on cancer immunotherapy with additional nucleotide-metabolic targets versus immunotherapy monotherapy from ClinicalTrials.gov. In total, 22 clinical trials were identified, and approximately 30% (7/22) of the studies were terminated or completed (Table 3). Therefore, most of the clinical trials are still in phase I/II and recruiting cancer patients from different cancer types and stages. Anti-PD-1 or anti-PD-L1 agents, including durvalumab, pembrolizumab, and nivolumab, are preferable choices for immunotherapy combined with nucleotide antimetabolites (Table 3). The included cancer patients have several “hot tumors,” such as NSCLC, melanoma, and hematological malignancies, with high sensitivity to immunotherapy [251, 252].

Among seven terminated or completed trials, only one trial has reported results [253, 254]. This phase I trial explored the efficacy and safety of NY-ESO-1T cells in synovial sarcoma patients; specifically, cohort 3 received immunotherapy plus only cyclophosphamide and cohort 4 received the same regimen with additional fludarabine (NCT01343043). The responses between these two cohorts did not seem to be significantly different. One important reason we considered was the difference in the dosage of cyclophosphamide (cohort 3: 1800 mg/m^2^/day × 3 days vs. cohort 4: 0.600 mg/m^2^/day × 3 days) [254].

In these currently undergoing and completed clinical trials, physicians attempted to compare the efficacy of additional therapeutic agents targeting nucleotide metabolism with immunotherapy versus immunotherapy monotherapy. Highly immune-sensitive tumors, such as NSCLC and melanoma, were preferable for inclusion. To our surprise, some cold tumors, such as TNBC and squamous lung cancer, were selected in further clinical trials.

Although much evidence from preclinical studies has demonstrated the potential application of additional nucleotide antimetabolites, the results of phase I/II clinical trials have indicated that physicians need to consider cautiously the selection of immunotherapy agents, nucleotide antimetabolites and included cancer patients to achieve the expectations. Furthermore, the accompanying incidence rates of adverse events may increase with the addition of chemotherapeutic agents. Therefore, clinicians must manipulate more health care for patients receiving immunotherapy with antimetabolites targeting nucleotide metabolism.

## Conclusion

The tight linkage between nucleotide metabolism and cancer immunity is discussed and summarized. Specifically, purine and its related metabolites, such as adenosine and ATP, are critical for the activation or suppression of innate and adaptive immune responses. Pyrimidine or purine metabolism dysregulation independently affects the pyrimidine versus purine ratio, whereas the ratio correlates with gene mutation and tumor immunogenicity. Furthermore, microbes can release nucleoside analogs to regulate the immune microenvironment, indicating potential effectiveness of fecal microbiota transplantation. Nucleotide metabolism is dynamically positioned within the cancer-immune cycle, and targeting this pathway proves the potential to enhance the effectiveness of immunotherapy. Targeting nucleotide metabolism in combination with immunotherapy agents could achieve promising synergistic effects on various cancers, warranting further validation in future clinical trials.

## Data Availability

Not applicable.

## Abbreviations

5-FU: Fluorouracil
ADA: Adenosine deaminase
ADSS: Adenylosuccinate synthetase
AML: Acute myeloid leukemia
AMP: Adenosine monophosphate
APCP: Adenosine 5'-(alpha, beta-methylene) diphosphate
ASOs: Antisense oligonucleotides
CAD: Carbamoyl phosphate synthetase, aspartyl transcarbamoylase, and dihydroorotase
CLK3: CDC-like kinase 3
CTLA-4: Cytotoxic T lymphocyte antigen 4
DHODH: Dihydroorotate dehydrogenase
DNMT: DNA methyltransferase
DPD: Dihydropyrimidine dehydrogenase
EMT: Epithelial-mesenchymal transition
ENTPD1/CD39: Ecto-nucleoside triphosphate diphosphohydrolase-1
FMT: Fecal microbiota transplantation
GART: Glycinamide ribonucleotide transformylase
GBM: Glioblastoma
GMP: Guanosine monophosphate
GMPS: Guanosine 5'-monophosphate synthase
HGPRT: Hypoxanthine–guanine phosphoribosyltransferase
hmdU: Nucleotide 5-hydroxymethyl-deoxyuridine
HNSCC: Head and neck squamous cell carcinoma
ICB: Immune checkpoint blockage
IMP: Inosine monophosphate
IMPDH: Inosine monophosphate dehydrogenase
MDSCs: Myeloid-derived suppressor cells
MM: Multiple myeloma
MPs: Monophosphates
mTORC1: MTOR complex 1
NFAT: Nuclear factor of activated T cells
NKG2D: Natural killer group 2D receptor
NLRs: NOD-like receptors
NSCLC: Non-small-cell lung cancer
NT4E/CD73: Ecto-5-nucleotidase
OMP: Orotidine monophosphate
PD-1: Programmed cell death protein 1
PNP: Purine nucleoside phosphorylase
POM-1: Sodium polyoxotungstate
PRPP: Phosphoribosyl pyrophosphate
PTMB: Purine-to-pyrimidine transversion mutations
RLRs: RIG-like receptors
RPIA: Ribose 5'-phosphate isomerase A
sAMP: Succinyl adenosine 5'-monophosphate
TAMs: Tumor-associated macrophages
TKI: Tyrosine kinase inhibitor
TLRs: Toll-like receptors
TMB: Tumor mutational burden
TME: Tumor microenvironment
TNBC: Triple-negative breast cancer
TYMP: Thymidine phosphorylases
UCK: Uridine–cytidine kinases
UDP: Uridine diphosphate
UHMK1: U2AF homology motif kinase 1
UMPS: Uridine monophosphate synthetase
UPP1: Uridine phosphorylase
XDH: Xanthine dehydrogenase
XMP: Xanthosine monophosphate
